# The Assessment of Early Glycosaminoglycan Concentration Changes in the Kidney of Diabetic Rats by Critical Electrolyte Concentration Staining

**Published:** 2013

**Authors:** Mohsen Pourghasem, Ebrahim Nasiri, Shima Sum, Hamid Shafi

**Affiliations:** 1 *Cellular and Molecular Biology Research Center, Babol University of Medical Sciences, Babol, Iran.*; 2 *Department of Anatomical Sciences, GilanUniversity of Medical Sciences, Rasht, Iran.*; 3 *Department of Social Medicin, Babol University of Medical Sciences, Babol, Iran.*; 4 *Department of Urology, Babol University of Medical Sciences, Babol, Iran.*

**Keywords:** Glycosaminoglycan, diabetes mellitus, kidney, CEC

## Abstract

Glycosaminoglycan (GAG) has a pivot role in renal function and homeostasis. Analysis of GAG amount generally serves to determine GAG alteration due to diabetes mellitus. Critical Electrolyte Concentration (CEC) staining can be an efficacy method to study GAG amount changes. Based on an experimental study, 20 male rats were randomly divided equally into two experimental and control groups. Diabetes mellitus was induced by a single sub cutaneous injection (120 mg/kg) of alloxan monohydrate. After 8 weeks, diabetic kidneys were paraffin embedded and sectioned at 5μm on a microtome. Slides were prepared and studied after staining by Critical Electrolyte Concentration (CEC 1 -4). In this study, we succeeded to show a decrease of hyaluronic acid and chondroitin sulfate concentration in diabetic kidney at 8 weeks diabetic rats which are earlier signs compared to those reported previously. In contrary, no significant changes in heparin sulfate and keratin sulfate have been seen. Diabetic nephropathy is a progressive disease and earlier diagnosis makes a better treatment design to reduce its development. CEC staining is able to determine degradation of hyaluronic acid and chondroitin sulfate synthesis in diabetic kidney of rats in an earlier time**.**

Diabetes mellitus is a metabolic disease due to disability in pancreas to either produce enough insulin or respond to insulin. Consequently, blood sugar will rise. The most common cause of chronic kidney failure and end-stage kidney disease is diabetic nephropathy. It is a progressive disease and leads to hemodialysis. Due to the important role of kidney in homeostasis, failure in renal function causes wide disorders in the body such as cardiovascular disease (1). Most researchers believe that the earliest detectable changes in the course of diabetic nephropathy will be seen 10 years after initiation of diabetes mellitus. Although, morphometric studies showed that the signs can be diagnosed 18 months after diabetes initiation (2). Vascular disorders involve both micro and macro vessels. Microangiopathy in kidney damages glomerular and tubular arteries. The primary place of filtration in the kidney is the wall of the glomerular capillary called glomerular filtration barrier (GFB) (3). The GFB is regulated to be selectively permeable. It does not allow free passage of macromolecules but is permeable to water and solutes. The property of GFB is related to its components including glomerular basement membrane, podocyte and endothelium cells. Function of glomerular basement membrane has been characterized to consist of many materials such as, Proteoglycans, collagen type IV, laminin and fibronectin  (4). 

Proteoglycans are hydrated molecules with properties established by their glycosaminoglycan chains as well as their core protein (5). They are produced in all cells of body and are secreted into the extracellular matrix or maintained at the cell surface. GAGs have an important role in the homeostasis of kidney. They introduce negative charges in GBM which are important for both the development of nephritic disorders and normal function of GFB. The decrease of glycosamino-glycans in diabetic mellitus patients leads to glomerular hyperfiltration and proteinuria(6).

Diabetic nephropathy is a progressive disease and earlier diagnosis makes a better treatment design to reduce its development. Several hypotheses were reported to explain renoprotective effect of GAGs in experimental diabetes such as down regulation of proteases, regulation of extracellular mesengial matrix synthesis and restoration of GBM anionic charges (7). Thus, this study was conducted to find a method to detect GAG changes of kidney at earlier stages of diabetes mellitus.

## Materials and Methods

Based on an experimental study, 20 male rats (wistar, weight 200 - 250 g) were randomly divided equally into two experimental and control groups. The animals were housed in standard laboratory condition, 12 hours light/darkness cycle, constant temperature, 50-55% moisture and easy access to food and water. Animal care was performed in accordance with Ethics Committee of Babol University of Medical Sciences.

Diabetes mellitus was induced by a single sub cutaneous injection (120 mg/kg) of freshly prepared solution of alloxan monohydrate (Aldrich, A7413-25G) dissolved in PBS (8). The induction of hyperglycemia was confirmed one week after treatment and reconfirmed the day of sacrificing using blood glucometer (Gluco care.77 Electronica kft, co) in rats with fasting using blood glucose levels above 200 mg/dl. Two rats were excluded due to blood glucose level lower than 200 mg/dl. Eight weeks after treatment, animals (both control and experi-mental groups) were anesthetized with ketamin (1.1mg/kg, ip.) before perfusion through the heart with 10% formaldehyde. 

The right kidney was dissected and immersed in 10% formaldehyde for 48 h. Then kidneys were paraffin embedded and sectioned at 5μm on a microtome. Slides were prepared and studied after staining with Critical Electrolyte Concentration (CEC 1 -4) and Hematoxilin – Eosin (H&E).

**Critical Electrolyte Concentration staining**

Alcian blue solution has been prepared with 0.5 gram alician blue in 1000 cc buffer acetate (0.2 M). For CEC1, 0.8 gram magnesium chloride in 100 cc alician blue solution was used. The a mounts of magnesium chloride for CEC2, CEC3 and CEC4 were 6.1, 10.1 and 18.3 respectively. Ten slides were randomly chosen from each kidney and incubated in CEC1-4 for 48 h (pH=5.8 at temperature 45-50 ^O^C). It had been taken cared of that each jur contained both the control and experimental slides. Alcoholic eosin was used as a counter stain. CEC 1, 2, 3, 4 determine hyaluronic acid, chondro-itin sulfate, heparin sulfate and keratin sulfate, respectively (9). Two blinded researchers quantified independently the concentr-ation of alcian blue according to a method described by Gong et al. with some modification  (10) using a light microscope (Olympus, Tokyo, Japan). The slides were scored from 1 to 4 according to increasing concentration of alcian blue. Thus, number one and four corresponded to the minimum and maximum concentration of alcian blue, respectively. Data analysis was carried out using SPSS soft ware (version 13). For the comparison of concentration of alcian blue between control and experimental groups, Mann-whitney test which is a non parametric test was performed and p-value <0.05 was considered as statistically significant.

## Results

To study the influence of diabetes mellitus on GAG concentration in rat kidneys, CEC staining which is a special method to determine tissue polyanions was used. For this purpose, the concentrations of salt (magnesium chloride) in alcian blue solution steadily increased and changed in staining occurred.

**Fig 1:**
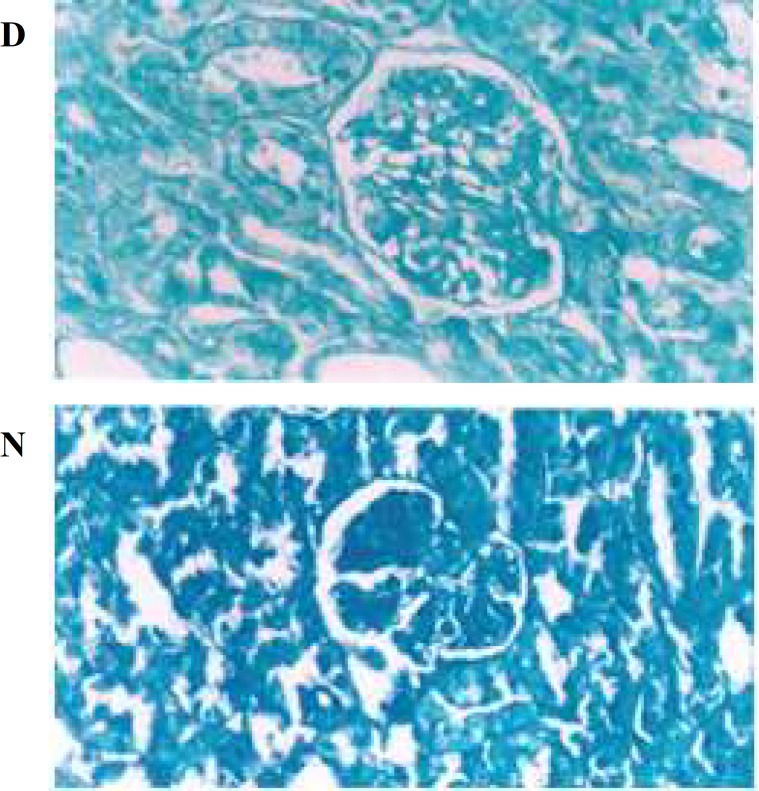
Decrease  of Alician blue concentration in diabetic kidney. D (diabetic kidney) and N (non diabetic kidney). CEC1× 200

**Fig 2:**
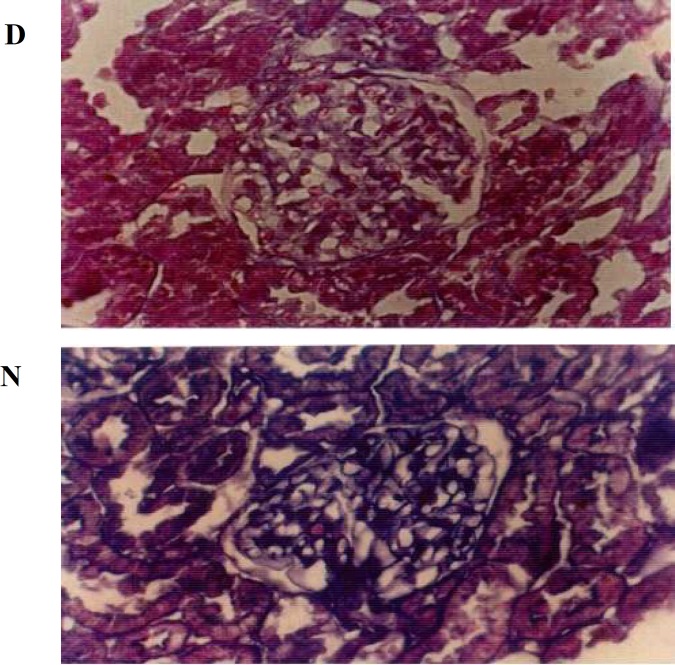
Decrease  of alician blue concentration in diabetic kidney. D (diabetic kidney) and N (non diabetic kidney). CEC2× 200

**graph 1:**
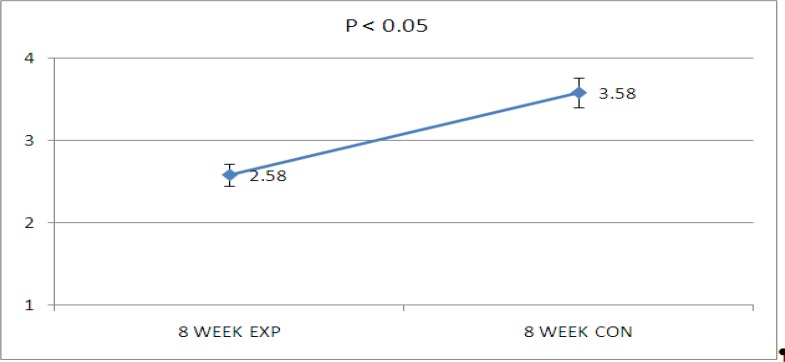
Decrease  of Alician blue concentration in diabetic kidney. D (diabetic kidney) and N (non diabetic kidney).CEC1× 200

**Graph 2:**
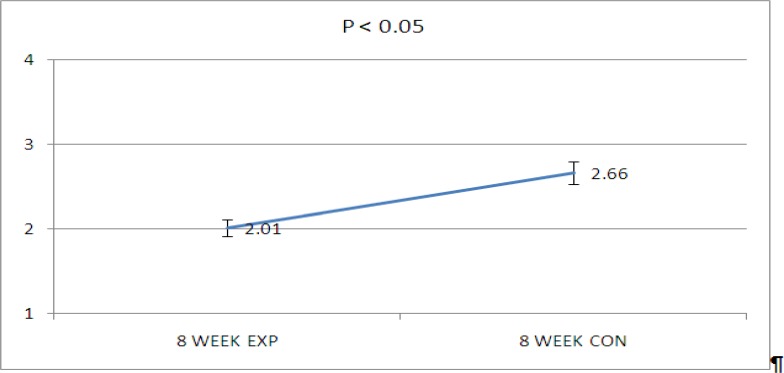
Decrease  of Alician blue concentration in diabetic kidney. D (diabetic kidney) and N (non diabetic kidney).CEC1× 200

Data showed that there were significant decrease of hyaluronic acid and chondroitin sulfate in diabetic kidneys while using CEC1 and CEC2, respectively in 8-week diabetic rats (P<0.05, Figures 1, 2 and Graph 1, 2). But  no significant changes have been seen in the concentration of alcian blue staining in CEC3 and CEC4. It means that there were no significant alteration in heparin sulfate and keratin sulfate concentration in this period of diabetes mellitus.

## Discussion

This study was conducted to find probable earlier GAGs changes in the kidney of diabetic rats using CEC staining. The mean of Critical Electrolyte Concentration is the distinctive concentration of salt at which a given polyanion ceases to stain with a given dye. On the other hand, the CEC of a polyanion –stain complex is a measure of the affinity of the stain for the polyanion, compared with that of a competition caution. The higher the CEC, the fewer substrates are stained (11). There is a reverse correlation between the amounts of GAGs and alcian blue concentration. It is clear that diabetic nephropathy is a progressive disease and quicker diagnosis romises better treatment and prognosis.

The roles of glycosaminoglycans in the pathogenesis of proteinuric kidney disease have been described extensively. The major classes of GAGs are hyaluronic acid, chondroitin sulfate, heparin sulfate and keratin sulfate. The determination of GAG changes in diabetes nephropathy has been limited to quantifying the amount of total GAG content. In this study, we succeeded to show the decrease of hyaluronic acid and chondroitin sulfate in diabetic kidneys at 8 weeks in the diabetic rats which are earlier signs compared to those reported (12). Although, diminished heparin sulfate has been reported in diabetes mellitus by counting anionic sites in electron micrographs (13)***,*** but we could not show the changes of heparin sulfate and keratin sulfate using CEC3 and CEC4, respectively. The reasons may be either there are no changes in the mentioned glycosaminoglycans in the short time or the amount of changes is not such to be distinguished by CEC method. Our data showed unequal changes in the amount of GAG among the diabetic kidneys.

The Steno hypothesis can explain the differences of the GAG amount alterations (14). The hypothesis hold that there is a genetic defect in the regulation of GAG synthesis in hyperglycemic situation. Deckert et al. previously reported the mesangial expansion in the diabetic rat (15) Apparently, there is a reverse correlation between mesangial proliferation and expression of glycosaminoglycans in diabetic nephropathy (16). Our own study showed similar changes in GAG content in the brain of the diabetic rats (17). It determines that the abnormalities in GAGs due to diabetes are not necessarily restricted to the kidney. GAGs bind and regulate many proteins which have important role in the function of kidney (18).These proteins include growth factors and their receptors, chemokines such as CXc and CC types, extra cellular components such as collagen and laminin, many enzymes such as lipase and protease. Therefore, reduced GAG amount induces important alterations in the renal function. Sulfating of the GAG has been disrupted in the hyperglycemic culture. Deckert et al. believe that changes in the GAG metabolism have pivotal role in vascular complication (15). It should be stressed that angiotensin 2 is increased in diabetes and has a negative control on production of transforming growth factor beta (TGF-B) and on the contrary, degradation of TGF-B inhibits GAG synthesis (19). Several reports indicated that GAG-like products treatment prevent and cure diabetic nephropathy in experimental diabetic animals (20).These materials may correct the balance between synthesis and degradation of Extra Cellular Matrix  content (21, 22). For example, sulodexide diminishes albuminuria and regulates matrix protein accumulation in diabetic mice (23, 24). In conclusion, our study shows that CEC staining is able to determine degradation of hyaloronic acid and chondroitinsulfate synthesis in diabetic kidney of rats in an earlier time. Regarding the similarity in the characteristic features of disease and pathological changes between rats and humans, the results may also come true in human kidney. Further investigations in diabetic patients may elucidate this hypothesis
